# Thermosensitive Chitosan Hydrogels Containing Polymeric Microspheres for Vaginal Drug Delivery

**DOI:** 10.1155/2017/3564060

**Published:** 2017-10-25

**Authors:** Ting-Ting Yang, Yuan-Zheng Cheng, Meng Qin, Yong-Hong Wang, Hong-Li Yu, An-Lin Wang, Wei-Fen Zhang

**Affiliations:** ^1^School of Pharmacy, Weifang Medical University, Weifang, Shandong 261031, China; ^2^Life Science and Technology Department, Pharmaceutical University, Nanjing, Jiangsu 211198, China

## Abstract

Thermosensitive hydrogels have increasingly received considerable attention for local drug delivery based on many advantages. However, burst release of drugs is becoming a critical challenge when the hydrogels are employed. Microspheres- (MS-) loaded thermosensitive hydrogels were thus fabricated to address this limitation. Employing an orthogonal design, the spray-dried operations of tenofovir (TFV)/*Bletilla striata* polysaccharide (BSP)/chitosan (CTS) MS were optimized according to the drug loading (DL). The physicochemical properties of the optimal MS (MS F) were characterized. Depending on the gelation temperature and gelating time, the optimal CTS-sodium alginate- (SA-) *α*,*β*-glycerophosphate (GP) (CTS-SA-GP) hydrogel was obtained. Observed by scanning electron microscope (SEM), TFV/BSP/CTS MS were successfully encapsulated in CTS-SA-GP. In vitro releasing demonstrated that MS F-CTS-SA-GP retained desirable in vitro sustained-release characteristics as a vaginal delivery system. Bioadhesion measurement showed that MS-CTS-SA-GP exhibited the highest mucoadhesive strength. Collectively, MS-CTS-SA-GP holds great promise for topical applications as a sustained-release vaginal drug delivery system.

## 1. Introduction

Vaginal delivery has advantages such as facility of drug application, high contact surface area, high blood supply, and avoidance of first-pass metabolism as a suitable route for systemic and topical drug delivery of antifungals, antibacterials, antivirals, and spermicidal agents [1, 2]. However, vaginal conventional dosage forms, including suppositories, lotions, pellicles, tables, gels, and irrigations, remain for only a short time and have uneven dispersion at the application site due to the physiological removal mechanisms of the vaginal lumen [3].

Thermosensitive hydrogels, intelligent materials endowed with a sol-gel phase transition in response to changes of temperature, have increasingly received considerable attention for local drug delivery based on many advantages including site-specificity, sustained-release behavior, simple drug formulation and administration, and improved safety [4]. The hydrogels are usually made from thermosensitive polymers such as poly(N-isopropylacrylamide) [5], polyethylene glycol [6], and chitosan (CTS) and its derivatives [7, 8].

CTS has a great deal of biomedical and pharmaceutical applications because of its distinctive characteristics such as biocompatibility, biodegradability, nontoxicity, and nonimmunogenicity [9–12]. Meanwhile, the degraded products of CTS are also nontoxic, nonimmunogenic, and noncarcinogenic [13]. CTS can allow better contact with the vaginal surface due to its mucoadhesive property [14]. CTS becomes thermoresponsive upon addition of *α*,*β*-glycerophosphate (GP), and the thermosensitive hydrogel (CTS-GP) has been reported by numerous literatures [8]. However, burst release of loaded drugs is becoming a critical challenge when the CTS-based hydrogels are employed as drug delivery systems [15]. To address this challenge, CTS-GP hydrogels containing drug-loaded microspheres (MS, MS-CTS-GP) begin to attract the attention [16]. CTS-GP-MS can localize the MS around the administrating target because the hydrogel can transform itself into semisolid gel at physiological temperature after administration. Then MS-CTS-GP continually release the drug from the double-component formulation, which further extends drug releasing time due to an auxiliary barrier of CTS-GP. So comparing with the single thermosensitive hydrogel and MS, the novel formulation simultaneously possesses triple abilities including localized drug delivery, long-term sustained drug release, and good biocompatibility [16]. Underlined by the advantages, MS-CTS-GP was supposed to be a promising formulation for mucosal drug delivery system, such as vaginal delivery systems. However, the studies on thermosensitive CTS hydrogels containing drug-loaded MS for vaginal delivery systems have been rarely conducted.

The present study mainly focused on the potential of this double-component MS-loaded thermosensitive hydrogel for vaginal delivery systems. Tenofovir (TFV)/*Bletilla striata* polysaccharide (BSP)/CTS MS were prepared by spray drying. TFV was a model drug to partially prevent HIV transmission to women [17]. BSP is a kind of viscous polysaccharide obtained from* Bletilla striata* and has various kinds of biological activities such as antibacterial, protection of gastric mucosa, hemostasis, antitumor, and promoting wound healing [18]. And BSP has been increasingly concerned in the research of drug doasge form because of the characteristics of biodegradability, biocompatibility [19–21]. BSP and CTS were used as pharmaceutical excipients to prepare microspheres by spray drying. Recently, sodium alginate (SA) has a great deal of biomedical and pharmaceutical applications because of its distinctive characteristics such as biocompatibility, biodegradability, and nontoxicity [22–24]. SA was mixed with CTS-GP solution to incorporate the mucoadhesive of CTS to obtain hydrogels (CTS-SA-GP) in this paper. Then the optimal MS were loaded into the CTS-SA-GP hydrogel to form a double-component formulation. The results for preparations and characterizations of TFV/BSP/CTS MS and MS-CTS-SA-GP were investigated in this study.

## 2. Materials and Methods

### 2.1. Materials

TFV (purity > 98%) was purchased from Biochempartner Company (Shanghai, China). To prepare hydrogels and MS, CTS (500 kDa and 300 cps, resp.) was obtained from Hai Debei Marine Biotechnology Company (Jinan, China), and the degree of deacetylation was 94% and 83%, respectively. BSP (650 kDa) was purchased from the Company of Huaqing Meicheng Natural Product Technical Development (Beijing, China). All other chemicals and solvents were reagents grade. Female rabbits were provided by Weifang Medical University. The animal protocol was approved by Shandong Medical Laboratorial Animal Administration Committee. The rabbits were housed in a room with controlled temperature and humidity. The animal study was carried out in accordance with the National Institutes of Health Guide for the Care and Use of Laboratory Animals (NIH publication number 8023) revised in 1978.

### 2.2. Preparation and Characterization of MS

#### 2.2.1. Preparation of MS

The L9(3^4^) orthogonal design was employed for optimization of spray-dried operation conditions. Inlet temperature, feed pump rate, the air-flow rate, and the ratio of TFV/BSP/CTS were selected as variables and studied at 3 levels as Table 1, and drug loading (DL) was taken as response parameter. Predetermined amounts of TFV, BSP, and CTS (500 KDa) were dissolved in 1000 mL of 0.5% acetic acid according to the predetermined orthogonal experimental design and were stirred vigorously for 8 h under room temperature. The solution was filtrated by 0.45 *μ*m micropore film, followed by spraying dried using a spray drier (Buchi, Mini Spray Dryer B-290, Flavil, Switzerland). The dried MS were stored in capped glasses container at 4°C for further analyses.

#### 2.2.2. DL and Entrapment Efficiency (EE) of MS

150 mg of MS was dissolved in distilled water and adjusted to 150 mL. Then a certain volume of the solution was sonicated for 5 min and kept overnight in orbital shaker to ensure TFV release completely. TFV contents of the MS were determined with a UV spectrophotometer (Shimadzu UV-Vis Spectrophotometer UV-1700, Japan) at 261 nm. The concentration of TFV was calculated using the equation as follows:

(1) $$\begin{array}{c} Y=25.64X-0.2154, \end{array}$$

where *X* is the absorbance value and *Y* is the concentration of TFV (*μ*g/mL). *R*^2^ is 0.9997.

The EE (%) and DL (%) were calculated using the following equations, respectively:

(2) EE %=Weight  of  drug  in  sampleTheoretical  weight  of  drug  in  sample×100%,DL %=Weight  of  drug  in  sampleWeight  of  sample×100%.

#### 2.2.3. Morphology of MS

The shape and surface morphology of MS were evaluated by scanning electron microscope (SEM) (TESCAN MAIA 3 LMH, Shanghai, China). The samples were mounted onto stubs using a double sided adhesive tape and were sputter-coated with gold prior to taking pictures.

#### 2.2.4. Size of MS

The mean volume diameters (MVDs) of MS were measured by dynamic light scattering (Nano ZS90, Malvern Instruments, Ltd., Mal vern, UK).

#### 2.2.5. Fourier Transformation Infrared Spectroscopy (FT-IR)

FT-IR analyses of pure TFV, BSP, CTS, BSP/CTS MS, and MS F were conducted using FT-IR Avater-360 Spectrometer (Nicolet, Madison, WI) in the range of 4000–500 cm^−1^.

#### 2.2.6. Thermal Analysis

Thermal analyses, including thermal gravity analysis (TG), differential thermal gravity (DTG), and differential Scanning calorimeter (DSC), of pure TFV, BSP, CTS, BSP/CTS MS, and MS F were conducted using simultaneous thermal analyzer (STA 449 F3 Jupiter®, Netstal, Germany) at heating rate of 20°C/min over a temperature range of 50–400°C.

#### 2.2.7. X-Ray Diffraction (XRD)

XRD patterns of pure TFV, BSP, CTS, BSP/CTS MS, and MS F were conducted using D8 Advance X-ray diffractometer (Brucker AXS, Germany) between 5° and 80° (2*θ*) at a scanning rate of 2° (2*θ*)/min.

### 2.3. Preparation and Characterization of MS-Loaded Hydrogel

#### 2.3.1. Preparation of MS-Loaded Hydrogel

2.00 g of CTS (300 cps) was dissolved in 100 mL of 100 mM acetic acid-sodium acetate buffer (pH, 4.5) and was stirred vigorously for 6 h under room temperature. 10% of calcium chloride (CaCl_2_), 50% of GP, and 0.5% of SA were prepared with distilled water. The following operations were at ice baths. The GP solution (15 mL) was carefully added into the CTS solution (35 mL) drop by drop to obtain a clear and homogeneous liquid solution. Then 10% CaCl_2_ solution (5 mL) was dropped into the above solution. Lastly 0.5% SA (6 mL) was dropped slowly. To form the MS-CTS-SA-GP complex hydrogels, suitable amount of MS F was dispersed in CTS-SA-GP hydrogels (labeled as MS F-CTS-SA-GP).

#### 2.3.2. Gelation of Thermosensitive Hydrogels

The gelation of thermosensitive hydrogel was determined by test tube inverting method [25, 26]. The gelation point was determined when the hydrogel could not flow over 30 s while the tube incubating in a water bath was inverted. If the gelation was not observed after 30 min, the temperature of the water bath would increase at the rate of 1°C each time in the range of 34 to 42°C. The effects of SA and MS on the viscosity of thermosensitive gel were analyzed by observing the time that the semisolid gel overcomes gravity to keep the gel from falling in plastic tubes, and the time was labeled as *T*_*k*_.

#### 2.3.3. Morphological Studies of Thermosensitive Hydrogels

The structural features of the hydrogel freeze-dried were observed by SEM (TESCAN MAIA 3 LMH, Shanghai, China). The samples were mounted onto stubs using a double sided adhesive tape and were sputter-coated with gold prior to taking pictures.

#### 2.3.4. In Vitro Drug Release

100 mg of MS F was put in dialysis bags (cutoff Mw 8000–14000), and 5 mL of 10 mM potassium phosphate buffer (pH 7.2) or 25 mM sodium acetate buffer (pH 4.5) was added. Then, the dialysis bags were loaded into a 200 mL flask containing 150 mL of the same release medium. Each sample was incubated in a constant temperature shaker at 37°C and a shaking speed of 100 rpm. At each defined time point, 500 *μ*L samples were pipetted and attenuated with the same buffer. The content of TFV was quantitated using UV spectrophotometer (Shimadzu UV-Vis Spectrophotometer UV-1700, Japan) at 252 nm. The release of free TFV was conducted under the similar condition.

The release of TFV from MS F-CTS-SA-GP was measured under predetermined conditions [26]. 1 mL of MS F-CTS-SA-GP was placed into a tube with inner diameter 10 mm. For the gel transformation, the tubes were incubated at 37°C for 20 min. Then, 10 mL of 10 mM potassium phosphate buffer (pH 7.2) or 25 mM sodium acetate buffer (pH 4.5) was added, respectively, followed by incubating in a constant temperature shaker at 37°C and a shaking speed of 100 rpm. At specific time points, 1 mL of medium was collected to quantitate the content of TFV while replacing with fresh buffer. The release of TFV from CTS-SA-GP was conducted under the similar condition.

#### 2.3.5. Bioadhesion Measurement

The ex vivo bioadhesiveness was studied according to the previously reported bioadhesion test [27]. Firstly, adhesive materials and vaginal mucosa of female rabbits were fixed on loading plates with 502 glue, respectively. Then the adhesive materials were pressed together with the vaginal mucosa, and this process needs to exert a certain amount of force for a certain period of time. After the adhesive bond has formed, the mucoadhesive strength was measured according to the weight of water required to separate the bond per square centimeter as shown in Figure 6(a).

#### 2.3.6. Statistical Analysis

The results were reported as mean ± standard deviations and each experiment was performed in triplicate. Statistical analysis was carried out using SPSS19.0 and differences were considered to be significant at a level of *P* < 0.05.

## 3. Results and Discussion

### 3.1. Optimization and Characterization of MS

#### 3.1.1. Optimization of Experimental Conditions

The optimization of MS was carried out with regard to DL as shown in Table 1. According to the *k* values, the optimal MS, MS F, was prepared as follows: the inlet temperature 130°C, the feed pump rate 3 mL/min, the air-flow rate 400 L/h, and the ratio of TFV/BSP/CTS 2 : 5 : 5. Additionally, according to the values of the range (*R*), the importance of the four factors was in the following order: the ratio of TFV/BSP/CTS > feed pump rate > inlet temperature > the air-flow rate, which indicated that the ratio of TFV/BSP/CTS has a larger effect on DL compared with the other three. When the ratio of TFV/BSP/CTS was 2 : 5 : 5, the DL was improved mostly, which might be due to the entrapping and adsorption capacity of CTS [28]. In addition, 3 mL/min of the feed pump rate resulted in the maximal value of DL. As for the influence of the inlet temperature, 130°C was the best spray condition, which could be attributed to that the optimum spray-drying thermal efficiency can be realized when the energy input and the energy needed achieve a balance [29]. And MS F prepared under the optimization of experimental conditions was endowed with the EE of 73.98% and the DL of 12.33%.

#### 3.1.2. Morphology and Particle Size

**Figure 1:**
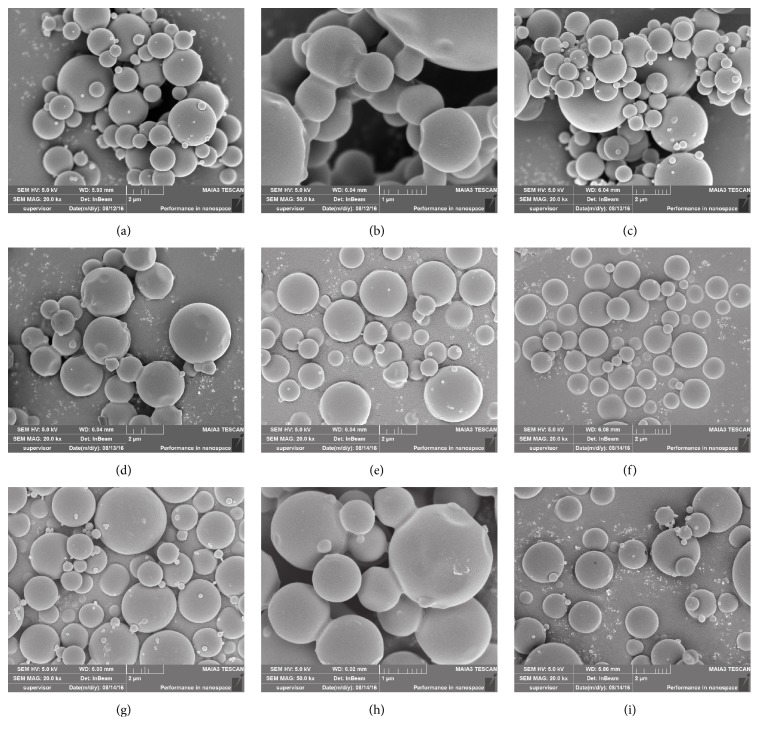
SEM of MS A (a), MS B (b), MS C (c), MS D (d), MS E (e), MS F (f), MS G (g), MS H (h), and MS I (i).

**Figure 2:**
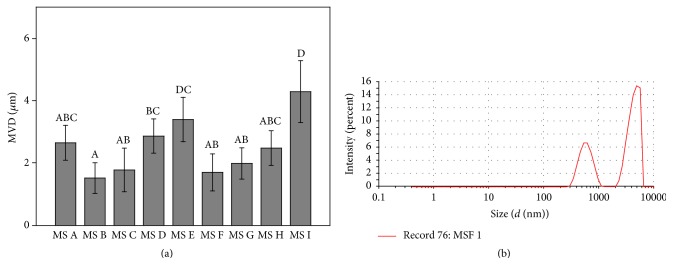
The geometric diameter determined via dynamic light scattering. (a) The MVDs of different MS. Bars represent standard errors of three replications, and different letters indicate significant differences according to Duncan's multiple range tests (*P* < 0.05); (b) The size distribution by intensity of MS F.

The morphological characteristics of the nine spray-dried microspheres were shown in Figure 1. MS A, C, E, F, G, and I showed nearly spherical shapes and smooth external surfaces, and these MS appear to be well dispersed. Adhesion phenomena were found in MS B and MS H. By varying instrumental parameters and solution concentrations, changes were also observed in terms of particle sizes. As shown in Figure 2(a), the MVDs ranged between 1.52 ± 0.49 *μ*m and 4.29 ± 0.99 *μ*m. The small CTS particles have higher binding capacity into the mucosa [30]. The MVD of MS F (1.70 ± 0.60 *μ*m) was larger compared with MS B (1.52 ± 0.49 *μ*m), but there was no significant difference between them (*P* > 0.05). And MS B was endowed with adhesion phenomenon, which could cause uneven dispersion in the hydrogel. The size distribution by volume of MS F was in the size range of 0.3–5 *μ*m in Figure 2(b). The large size range distribution was probably because of different evaporation rates of water in prescription solution with pumping.

#### 3.1.3. FT-IR Analysis

**Figure 3:**
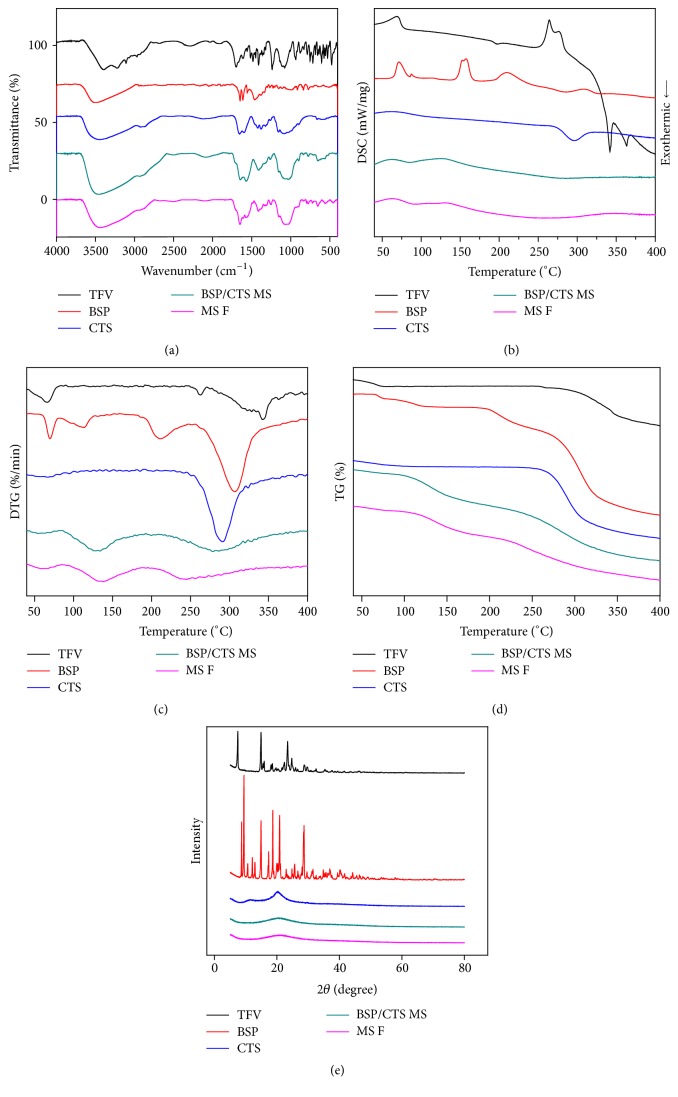
Spectra analyses of TFV, BSP, CTS, BSP/CTS MS, and MS F. (a) FT-IR spectra; (b) DSC spectra; (c) DTG; (d) TG spectra; (e) XRD spectra.

The FT-IR spectra of TFV, BSP, CTS, BSP/CTS MS, and MS F were shown in Figure 3(a). In FT-IR spectrum of BSP, the strong and broad absorption band at 3505 cm^−1^ was assigned to hydroxyl group stretching vibrations. The bands at 1647 cm^−1^ and 1612 cm^−1^ were assigned to stretching vibration of C=O in carbonyl groups and bound water, respectively [31]. Compared with BSP, the peak of hydroxyl group stretching vibrations in the spectra of BSP/CTS MS was much broader with a shift to lower frequency about 50 cm^−1^ (3505 → 3455 cm^−1^). Meanwhile, the strong characteristic peak at 1650 cm^−1^ belonging to the vibration of C=O of CTS shifted to a lower frequency side (1655 → 1645 cm^−1^), which indicated the formation of intermolecular hydrogen bonds between the carbonyl group and the hydroxyl group of BSP. With the addition of TFV, the peak of hydroxyl group stretching vibrations shifted to a further lower frequency by about 12 cm^−1^, which may be due to the formation of intermolecular hydrogen bonds between TFV and the used excipients. This was manifested by the blue shifts of the peaks of amide carbonyl (1698 cm^−1^) presented in pure TFV.

#### 3.1.4. Thermal Analysis

The DSC, DTG, and TG curves of pure TFV, BSP, CTS, BSP/CTS MS, and MS F were shown in Figures 3(b), 3(c), and 3(d), respectively. In the DSC thermograph, TFV exhibited a broad endothermic peak in approximate temperatures of 70°C due to the release of water from TFV. The corresponding mass loss near 70°C was observed in spectrums of DTG and TG, respectively. Following the water evaporation, an exothermic peak occurred around 197°C in the DSC thermograph of TFV because of recrystallization of tenofovir [32]. Subsequently, the peaks corresponding to the melting and exothermic degradation were shown at approximately 260°C and above 300°C, respectively. As shown in Figure 3(c), there were two peaks of mass loss at 70°C and 110°C for BSP, respectively, which were related to the loss of adsorbed and structural water in BSP sample [33]. The mass loss near 310°C was the characteristic feature of polysaccharide, coinciding with the DSC and TG analyses. CTS showed an exothermic peak above 290°C attributed to the side-chain degradation in the DSC thermograph [34], which was supported by the DTG and TG curves. As shown in the DSC thermograph of BSP/CTS MS, the two endothermic peaks attributed to the loss of adsorbed and structural water in BSP sample were broader and lower compared with that in the DSC thermograph of BSP, which could be because of spray drying. And the characteristic degradation process of polysaccharide was observed at temperature of 280°C lower than the degradation stage of CTS and BSP, indicating that the BSP/CTS MS was less stable than CTS and BSP. The instability of BSP/CTS MS could be as a result of the formation of intermolecular hydrogen bonds destroying the original crystalline structures [35]. But observing the relevant peaks in the DTG and TG thermographs, respectively, the mass loss of BSP/CTS MS was lower than that of BSP and CTS, demonstrating a reduction in degradation degree. With the addition of TFV, the degradation process of polysaccharide appeared at temperature of 240°C further lower than the degradation stage of BSP/CTS MS, which could also result from the formation of intermolecular hydrogen bonds. The melting of TFV occurring around 260°C was absent, indicating large amount of loaded TFV was in the amorphous form relative to pure TFV powder. And there was no peak corresponding exothermic degradation of TFV in the DSC thermograph of MS F, which was in line with the lack of relevant mass losses peaks in the DTG and TG thermographs and demonstrating that there were not only simple physical mixtures, but also new chemical bonds which influence chemical thermal stability.

#### 3.1.5. XRD Analysis

The XRD patterns of TFV, BSP, CTS, BSP/CTS MS, and MS F were shown in Figure 3(e). The characteristic crystalline peaks of pure TFV were observed at the diffraction of 2*θ* = 7.46°, 14.84°, 18.16°, 22.28°, 23.55°, 24.56°, 28.46°, and 29.42°. The diffraction curve of BSP contained many sharp peaks, indicating the BSP was crystalline material. The CTS exhibited two major crystalline peaks at 11.54° and 20.24°. However, the crystalline peaks of BSP and CTS entirely disappeared, and an amorphous state was present in the BSP/CTS MS, suggesting that the formation of chemical bonds between CTS and BSP suppressed the crystallization of CTS and BSP, and corresponded to the results of FT-IR analysis and thermal analysis. With the addition of TFV, there were still no crystalline peaks observed, indicating that TFV entrapped into the MS was in the amorphous state. This result was supported by the thermoanalysis showing that the melting of TFV was absent.

### 3.2. Optimization and Characterization of MS-Loaded Thermosensitive Hydrogels

#### 3.2.1. Optimization of Thermosensitive Hydrogels

**Figure 4:**
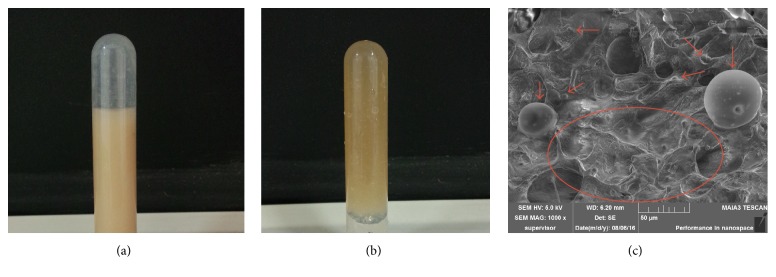
Sol state (a), gel state (b), and SEM (c) of MS F-CTS-SA-GP. MS F were loaded on CTS-SA-GP hydrogel and indicated by red arrows and circles (c).

The thermosensitive hydrogels were prepared by the method described in the previous item. When MS F was dispersed in CTS-SA-GP hydrogels, the solution changed from clear and homogeneous to brown and opaque (Figure 4(a)). And the solution became nonflowing gels at 37°C (Figure 4(b)).

Dependences of the gelating temperature, time, and viscosity on the different compositions in formulations were shown in Table 2. The different formulations containing 6 mL and 0 mL of SA, respectively, had the gelation times of 17.0 ± 1.0 and 18.5 ± 1.5 min, respectively, indicating that the the addition of SA in a certain range had no significant effect on the gelating time (*P* > 0.05). But it could be seen that the addition of SA could remarkably prolong the time when the semisolid gel overcomes gravity to keep the gel from falling in plastic tubes (*P* < 0.05). This was because SA could strengthen the viscosity through forming polyelectrolyte with CTS by electrostatic interactions [36], and the stronger viscosity in semisolid state could help overcoming the physiological removal mechanisms of the vaginal lumen, increasing the absorption of drugs. With the addition of MS F, the gelating time of MS F-CTS-SA-GP was 6.5 ± 1.5 min, which was significantly reduced comparing with CTS-GP and CTS-SA-GP (*P* < 0.05). Meanwhile, the addition of MS F could extend the time of keeping the gel from falling in plastic tubes comparing with the formulation B (*P* < 0.05), which increasing the absorption of drugs.

#### 3.2.2. Morphological Studies of Hydrogels

The surface morphological studies of MS F-CTS-SA-GP were observed in Figure 4(c). The hydrogel exhibited irregular porous three-dimensional structure, which facilitates entrance of water into the hydrogel networks and helps MS to diffuse out of them. As shown in Figure 4(c), the incorporation of MS F did not change porous microstructures of thermosensitive chitosan hydrogels [26]. And it was clear that MS F not only was well dispersed in the porous structure of CTS hydrogels in random form but also stayed spherical after the incorporation.

#### 3.2.3. In Vitro Drug Release

**Figure 5:**
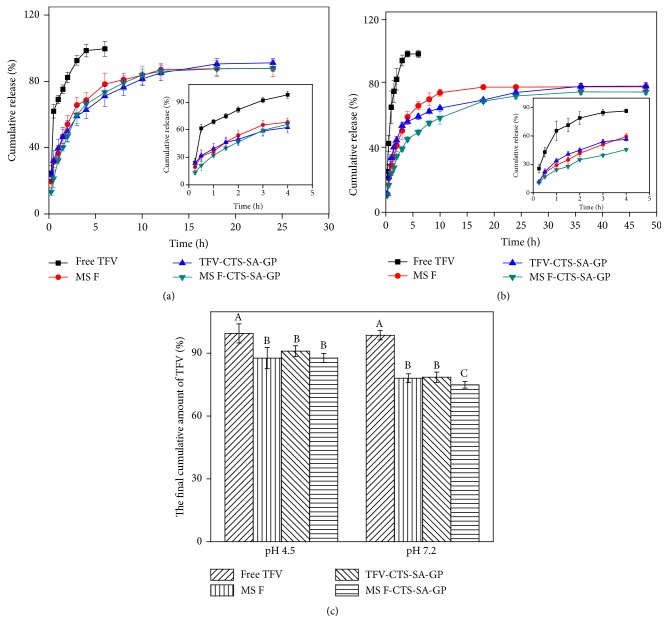
The in vitro time-dependent TFV release profiles of free TFV, TFV-CTS-SA-GP, MS F, and MS F-CTS-SA-GP at pH 4.5 (a) and pH 7.2 (b); (c) the final cumulative amount of TFV from different dosage forms at pH 4.5 and pH 7.2; different letters indicate significant differences according to Duncan's multiple range tests (*P* < 0.05).

The in vitro time-dependent TFV release profiles of free TFV, TFV-CTS-SA-GP, MS F, and MS F-CTS-SA-GP were shown in Figure 5. TFV, a small hydrophilic molecule drug, has reasonably speculated that a dialysis membrane would not interfere with its release [37], which was consistent with the experimental results that free TFV diffused completely through the dialysis membrane in about 4 h, both at pH 4.5 and at pH 7.2. As shown in Figure 5(a), in 25 mM sodium acetate buffer (pH 4.5) simulated vaginal environment, the release of TFV from MS F showed an initial burst release of more than 30% after 0.5 h and finally achieved the cumulative release of 87.82% (>75%) after 24 h. The similar explosive discharge of TFV in CTS-SA-GP group was also observed, which could be because of the hydrophilic microenvironment in the interconnecting pore structures produced during the process of sol-gel phase transition [16]. When embedding MS F into CTS-SA-GP, the cumulative amount of TFV released from MS F-CTS-SA-GP was about 13%, 21%, and 32% after 0.25, 0.5, and 1 h, respectively, displaying much slower TFV release rate without initial burst release compared with MS F and TFV-CTS-SA-GP within an hour. The slower TFV release rate might be due to an additional diffusion barrier provided by the gel matrix for the surface adsorbed drug of MS F. The final cumulative amount of TFV from MS F-CTS-SA-GP was lower than free TFV (*P* < 0.05) as shown in Figure 5(c). However, compared with the single-component formulations, there was no significant difference on the final cumulative amount of TFV according to Duncan's multiple range tests (*P* > 0.05) at pH 4.5. These results indicated that the double-component formulation did not affect drug dissolution at pH 4.5 and retained desirable in vitro controlled release characteristics as an effective vaginal delivery system.

As found at pH 4.5, the drug releases from the three formulations had the same tendency in the 10 mM potassium phosphate buffer (pH 7.2) simulated physiological condition (Figure 5(b)). And comparing with release profiles at pH 4.5, the cumulative release rate of TFV from TFV-CTS-SA-GP or MS F-CTS-SA-GP at pH 7.2 was much slower, which might be attributed to the slow dissolution of CTS in neutral medium. The final cumulative amount of TFV from MS F-CTS-SA-GP was about 75%, which was remarkably lower than MS F and CTS-SA-GP as shown in Figure 5(c) (*P* < 0.05), indicating that the TFV dissolution was affected when MS F-CTS-SA-GP was acted as a formulation in neutral medium (pH 7.2) compared with TFV-CTS-SA-GP and MS F.

TFV release kinetics from MS F, TFV-CTS-SA-GP, and MS F-CTS-SA-GP were analyzed using known release kinetic models including zero-order equation, first-order equation, Higuchi model, Ritger-peppas model, and Weibull model. The determination coefficients (*R*^2^) calculated by these models were summarized in Table 3. Considering the *R*^2^ values, the Weibull equation could better fit the TFV release curves in the three different formulations both at pH 4.5 and at pH 7.2. The constant *b* values of Weibull equation are in connection with the diffusional mechanism. It was concluded that the TFV release from MS F, TFV-CTS-SA-GP, and MS F-CTS-SA-GP followed Fickian diffusion at pH 4.5 and pH 7.2 because the values of *b* were all lower than 0.75 [38]. For Fickian diffusion, the disorder of the medium in the MS F-CTS-SA-GP or MS F group was lower than that in TFV-CTS-SA-GP group both at pH 4.5 and at pH 7.2 by observing values of *b* [38].

#### 3.2.4. Bioadhesion Measurement

**Figure 6:**
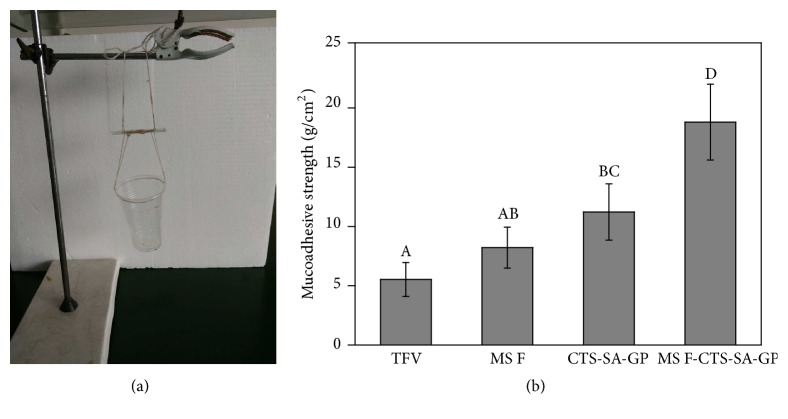
Bioadhesion measurement including the experimental arrangement (a) and the results of mucoadhesive strength (b) (*n* = 3), and different letters indicate significant differences according to Duncan's multiple range tests (*P* < 0.05).

Bioadhesion is a crucial parameter for in situ gels of vaginal formulation [39]. The bioadhesion measurement of TFV, MS, CTS-SA-GP, and MS F-CTS-SA-GP is shown in Figure 6(b). On the whole, the mucoadhesive strength of the four groups was in the following order: MS F-CTS-SA-GP (18.45 ± 3.14 g/cm^2^) > CTS-SA-GP (11.00 ± 2.33 g/cm^2^) > MS F (8.06 ± 1.7 g/cm^2^) > free TFV (5.42 ± 1.40 g/cm^2^). According to Duncan's multiple range tests, the bioadhesion of CTS-SA-GP and MS F-CTS-SA-GP was significantly improved comparing with TFV (*P* < 0.05), suggesting that thermosensitive hydrogels and thermosensitive hydrogels containing drug-loaded microspheres could both significantly prolong the vaginal residence time of TFV. And the bioadhesion of MS F-CTS-SA-GP was significantly better than that of MS F and CTS-SA-GP (*P* < 0.05), indicating that this double-component MS-loaded thermosensitive hydrogel exhibited the highest mucoadhesive strength, and both MS F and CTS-SA-GP had a major role in the preferable mucoadhesive strength for vaginal drug delivery.

## 4. Conclusions

In this study, TFV/BSP/CTS MS were successfully prepared and optimized to obtain the maximum possible DL. The importance of the four factors on DL was in the following order: the ratio of CTS/BSP/TFV > feed pump rate > inlet temperature > the air-flow rate. Under the optimal experimental conditions, MS F was endowed with the EE of 73.98% and the DL of 12.33%. MS F appearing to be nearly spherical in shape and to have smooth external surfaces was well dispersed with the MVD of 1.70 ± 0.60 *μ*m in size range of 0.3–5 *μ*m. The results of FT-IR, thermoanalysis, and XRD indicated the formation of intermolecular hydrogen bonds, the higher degradation temperature of TFV in TFV/BSP/CTS MS comparing with pure TFV powder, and the crystal transition of TFV from crystalline state to amorphous state. The addition of a certain amount of SA had no significant effect on the gelating temperature and time but could strengthen the viscosity of CTS-GP. MS F-CTS-SA-GP had less gelating time and stronger viscosity comparing with CTS-SA-GP. Observed by SEM, the irregular porous three-dimensional structure of the thermosensitive chitosan hydrogels was not changed with the incorporation of MS F. MS F remained spherical and well dispersed in the porous structure of the hydrogel. The release of TFV from MS F-CTS-SA-GP was without initial burst release compared with MS F and TFV-CTS-SA-GP. And at pH 4.5, the final cumulative amount of TFV was not affected by the double-component formulation. And the double-component formulation exhibited the preferable mucoadhesive strength. Taken together, thermosensitive chitosan hydrogels containing polymeric microspheres have the potential to be an appropriate formulation for sustained-release vaginal delivery system.

## Figures and Tables

**Table 1:** The L9(3^4^) orthogonal design for the optimization of spray-dried operation conditions.

Formulation	Factors				Results	
	*A* ^a^	*B* ^b^	*C* ^c^	*D* ^d^	DL, %	EE, %
MS A	120	8	400	1 : 5 : 5	6.73 ± 0.51	74.03 ± 5.61
MS B	120	5	500	2 : 5 : 5	9.10 ± 0.71	54.60 ± 4.26
MS C	120	3	600	1 : 2.5 : 5	9.47 ± 0.35	80.50 ± 2.98
MS D	130	8	500	1 : 2.5 : 5	10.92 ± 0.42	92.82 ± 3.57
MS E	130	5	600	1 : 5 : 5	6.80 ± 0.22	74.80 ± 2.42
MS F	130	3	400	2 : 5 : 5	12.33 ± 0.75	73.98 ± 4.50
MS G	140	8	600	2 : 5 : 5	9.19 ± 0.55	55.14 ± 3.30
MS H	140	5	400	1 : 2.5 : 5	9.69 ± 0.50	82.37 ± 4.25
MS I	140	3	500	1 : 5 : 5	8.69 ± 0.49	95.59 ± 5.39
*k* ^e^1	8.43	8.94	9.58	7.41		
*k*2	10.01	8.53	9.57	10.20		
*k*3	9.19	10.16	8.49	10.03		
*R* ^f^	1.58	1.63	1.09	2.79		
Important order	*D* > *B* > *A* > *C*					
The optimum condition DL	*A*2	*B*3	*C*1	*D*2		

*A* ^a^, *B*^b^, *C*^c^, and *D*^d^ represent four variables, respectively, as follows: inlet temperature, °C; feed pump rate, mL/min; the air-flow rate, L/h; the ratio of TFV/BSP/CTS (W/W/W); *k*^e^ show the relationship between the factors and the value of DL; *R*^f^ was used to determine the important order of experiment factors on the values of DL by means of range analysis.

**Table 2:** The effect of SA and MS F on the gelating temperature, time, and *T*_*k*_ of keeping the gel from falling in plastic tubes.

Formulation	SA, mL	MS F, mg/mL	Gelation		*T*_*k*_
			Temperature, °C	Time	
A	0	0	37	18.5 ± 1.5a^a^	4.6 ± 3.0a^a^
B	6.0	0	37	17.0 ± 1.0a^a^	14.2 ± 5.9b^b^
C	6.0	9.85	37	6.5 ± 1.5b^b^	26.0 ± 4.0c^c^

a^a^, b^b^, and c^c^ are the analysis results of Duncan's multiple range tests, and the different letters indicate significant differences (*P* < 0.05).

**Table 3:** Kinetic models used for analysis of TFV release rate from MS F, TFV-CTS-SA-GP, and MS F-CTS-SA-GP and their corresponding *R*^2^ values.

Formulations	Model name	Model equation	*R* ^2^	
			pH 4.5	pH 7.4
MS F	Zero-order	*Q* = *α* + *βt*	0.6455	0.6392
	First-order	ln⁡(1 − *Q*) = *α* + *βt*	0.8104	0.7558
	Higuchi	*Q* = *α* + *βt*_1/2_	0.8477	0.9489
	Ritger-peppas	ln⁡*Q* = *α* + *β*ln⁡*t*	0.9301	0.8934
	Weibull	ln⁡(−ln⁡(1 − *Q*)) = ln⁡*α* + *β*ln⁡*t*	0.9771 (*b*^a^ = 0.6221)	0.9905 (*b*^a^ = 0.5304)

TFV-CTS-SA-GP	Zero-order	*Q* = *α* + *βt*	0.7522	0.5288
	First-order	ln⁡(1 − *Q*) = *α* + *βt*	0.9289	0.7009
	Higuchi	*Q* = *α* + *βt*_1/2_	0.9218	0.7467
	Ritger-peppas	ln⁡*Qn* = *α* + *β*ln⁡*t*	0.8698	0.8382
	Weibull	ln⁡(−ln⁡(1 − *Q*)) = ln⁡*α* + *β*ln⁡*t*	0.9961 (*b*^a^ = 0.5003)	0.903 (*b*^a^ = 0.4178)

MS F-CTS-SA-GP	Zero-order	*Q* = *α* + *βt*	0.6556	0.6392
	First-order	ln⁡(1 − *Q*) = *α* + *βt*	0.9289	0.7558
	Higuchi	*Q* = *α* + *βt*_1/2_	0.8487	0.9489
	Ritger-peppas	ln⁡*Q* = *α* + *β*ln⁡*t*	0.8698	0.8934
	Weibull	ln⁡(−ln⁡(1 − *Q*)) = ln⁡*α* + *β*ln⁡*t*	0.9644 (*b*^a^ = 0.6289)	0.9905 (*b*^a^ = 0.5077)

*b* ^a^ is the constant of Weibull models.
